# Win-stay and win-shift lever-press strategies in an appetitively reinforced task for rats

**DOI:** 10.3758/s13420-016-0225-2

**Published:** 2016-08-18

**Authors:** Phil Reed

**Affiliations:** Department of Psychology, Swansea University, Singleton Park, Swansea, SA2 8PP UK

**Keywords:** Spontaneous alternation, Win-shift/lose-stay, Win-stay/lose-shift, Conditioning chamber, Food, Rats

## Abstract

Two experiments examined acquisition of win-stay, win-shift, lose-stay, and lose-shift rules by which hungry rats could earn food reinforcement. In Experiment 1, two groups of rats were trained in a two-lever operant task that required them to follow either a win-stay/lose-shift or a win-shift/lose-stay contingency. The rates of acquisition of the individual rules within each contingency differed: lose-shift and lose-stay rules were acquired faster than win-stay and win-shift rules. Contrary to a number of previous reports, the win-shift rule was acquired less rapidly than any of the other rules. In Experiment 2, the four rules were taught separately, but subjects still acquired the win-shift rule more slowly than any of the other rules.

When faced with a choice in a maze, rats typically enter unvisited arms in preference to those in which they have been reinforced previously, and this phenomenon is sometimes referred to as “spontaneous alternation” (e.g., Dember & Richman, 2012; Olton, Collison, & Werz, 1977; Timberlake & White, 1990). Several reports have noted that rats will more readily display such an appetitively reinforced alternation rule (i.e., a win-shift rule) than a win-stay rule, in a number of apparatus, such as the T-maze (e.g., Cohen, Westlake, & Szelest, 2004; Stanton, Thomas, & Brito, 1984), complex mazes (e.g., Montgomery, 1951), the Meir three-table task (Olton, 1982), and the radial maze (e.g., Olton & Samuelson, 1976; Olton & Schlosberg, 1978). It has been suggested that this tendency to alternate reflects an inherent foraging strategy for the rat (Charnov, 1976; Krebs & McCleery, 1984), which is based on a presumed need to avoid patch depletion (see Rayburn-Reeves, Stagner, Kirk, & Zentall, 2013). There are, of course, demonstrations that rats can acquire win-stay rules in maze apparatus when these contingencies are in place, but, when there are no constraints, it is assumed that they will typically show win-shift performance (see Cohen et al., 2004).

Although the results from maze-based studies provide strong evidence for spontaneous alternation in the rat, the results from reports using conditioning chambers are more ambiguous (cf. Evenden & Robbins, 1984; Morgan, 1974; Rayburn-Reeves et al., 2013). Of course, rats will learn win-stay behavior very readily in the operant chamber when this is explicitly taught, but it is unclear whether this behavior, or a win-shift strategy, is more readily adopted under conditions of no constraint. In this context, Morgan (1974) reinforced rats for pressing either of two levers that had equal frequencies of reward. In this study, subjects responded more often to the lever that had just been reinforced, and tended to shift levers if the last response was not rewarded (see also Rayburn-Reeves et al., 2013; Williams, 1991, for examples using slightly different procedures in the conditioning chamber). In contrast, Evenden and Robbins (1984; Experiment 2) demonstrated that win-shift behavior did emerge and persist over long periods of time in an operant conditioning chamber. However, although win-shift behavior was obtained by Evenden and Robbins (1984), they did not report the extent to which subjects exhibited lose-shift behavior, and it is possible that lose-shift behavior would also have been exhibited by subjects in the Evenden and Robbins (1984) report, perhaps even to a greater extent than win-shift behavior. In fact, there are almost no data relating to the comparative speed of acquisition of win- and lose-based rules under such conditions. Rayburn-Reeves et al. (2013) did note that rats tended to adopt a win-stay strategy during within-session serial reversal training, but this design did not provide scope for a comparative analysis of the rate of this win-stay behavior with win-shift behaviors.

In addition to the apparent inconsistency in the results reported from tasks in the conditioning chamber (cf. Evenden & Robbins, 1984; Morgan, 1974), and the lack of evidence relating to the speed acquisition of various rules, another hindrance to the comparison of processes responsible for spontaneous alternation in various apparatus exists. Reports of spontaneous alternation in the T-maze, for example, typically force the subjects to either one of the alternative arms, and then the subjects have to perform according to the specified reinforcement rule when faced with the two alternative arms at some later time (see Cohen et al., 2004; Olton, 1979, for various variations in this procedure). This procedure is not typically employed in the conditioning chamber. Rather, studies in the conditioning chamber have typically not required rats to perform according to a specific contingency (e.g., Evenden & Robbins, 1984; Morgan, 1974; Williams, 1991). This difference in the assessment of performance may be responsible (in some unspecified manner) for the different results with respect to the predominance or not of win-shift or win-stay behavior in the different apparatus.

The present report attempted to examine the relative speed of acquisition of the four forms of reinforcement rule (win-stay, win-shift, lose-stay, and lose-shift) by rats in the conditioning chamber. It attempted to address the above problems by studying alternation behavior in a conditioning chamber but employing the procedures typically used during T-maze studies of spontaneous alternation. That is, the participants were presented with an initial trial (i.e., they responded to a particular lever) that either did or did not result in reinforcement, and then they were presented with both levers and received reinforcement for pressing one of the levers depending on the reinforcement rule in operation (i.e., for pressing the same lever that was just reinforced in a win-stay trial, etc.). This more discrete-trial approach, rather than noting the free-operant behavior of rats, has closer parallels to the discrete trials often used in T-maze procedures. Such an approach may help to clarify the nature of the effects observed in spontaneous alternation studies, and any differences in the results obtained from the different apparatus used to highlight a range of potential theoretical implications regarding why these differences might occur.

## Experiment 1

The first experiment trained rats in a two-lever conditioning chamber. One group of rats obtained reinforcement according to a win-stay/lose-shift contingency, and the other group according to a win-shift/lose-stay contingency. For both of these contingencies, the outcome of the first half of the trial, in conjunction with the contingency in operation, would determine the response to be reinforced in the second half of the trial. This procedure offers a formal replication of that used for non-spatial discrimination in the T-maze.

### Method

#### Subjects

Forty-eight experimentally naïve, male Long Evans hooded rats served in the present experiment. The rats were 3–4 months old at the start of training, had a free-feeding body weight of 275–380 g, and were maintained at 85 % of this weight throughout the study. The subjects were housed individually, and had constant access to water in the home cage.

#### Apparatus

Eight identical operant conditioning chambers (Campden Instruments Ltd., Loughborough, UK) were used, measuring 30 cm high × 30 cm front to back × 45 cm long (back wall to levers). Each chamber was housed inside a light and sound attenuating case. A 65-dB(A) background masking noise was supplied by a ventilating fan. Each chamber was equipped with two retractable levers positioned 15 cm apart. The food tray into which reinforcement (one 45-mg food pellet) could be delivered was covered by a clear hinged, Perspex flap, and was centrally located between the two levers. A light could be operated to illuminate the magazine tray on delivery of reinforcement. A house light was located centrally on the same chamber wall as the magazine tray and two response levers.

#### Procedure

The rats received one session of training per day. The subjects initially were magazine trained in two 40-min sessions, during which food pellets were delivered according to a random time (RT) 60-s schedule. For the first session, the flap covering the magazine tray was taped open to allow easy access to the pellets. During the second session, the flap was lowered to its standard resting position. Subjects were then trained to lever press by reinforcing every response (i.e., a continuous reinforcement [CRF] schedule). Two sessions of CRF were given, one on each lever, and each session lasted until 75 reinforcements had been earned. Subjects were then given four 30-min sessions of a random interval (RI) 60-s schedule (two sessions on each lever). During these sessions, only one lever was present in the chamber. Following this training, subjects received two 30-min sessions during which both levers were inserted into the chamber, and responses to either could earn reinforcement. Reinforcement was delivered for responses to each lever according to independent RI 60-s schedules (i.e., a concurrent RI-60s, RI-60s schedule was in force). On delivery of food, the interval requirement on both levers was reset. Two rats failed to respond after this pretraining and were excluded from the study. The rats were then divided into two equal-sized groups (*n* = 23), matched for response on the two levers over the two sessions of concurrent schedule training.

During the critical experimental phase, rats in the two groups were treated identically to each other except for the rule that earned reinforcement. During the inter-trial interval (ITI), which lasted 30 s, the house light was off, and both levers were retracted from the chamber. Each trial consisted of two elements: an information stage and a choice stage. The house light was illuminated throughout each trial. For the information stage, one lever was randomly selected, and presented for 15 s. If the rat did not fulfil the response requirement (see below), the lever was withdrawn, the trial was abandoned, and the ITI of 30 s commenced. If the rat completed the response requirement within the specified time, the lever was withdrawn and the trial continued. The response requirement was one response for Sessions 1 and 2, two responses for Sessions 3 and 4, and three responses for the remainder of the experiment. In addition to the lever withdrawal, completion of the response requirement sometimes led to the delivery of a food pellet (on a win trial), and sometimes did not (on a lose trial). On win trials, the food was delivered, and the tray light was illuminated for 1 s. Following this, the tray light was turned off, and both levers were inserted into the chamber for the choice stage. On lose trials, no reward was delivered and no tray light illumination occurred, but after 1 s following the completion of the information stage and withdrawal of the lever, both levers were presented to the subject for the choice stage.

The identity of the correct lever during the choice stage was determined by a combination of the identity of the lever presented in the information stage, and the outcome of the response. During the choice stage, rats in the win-shift/lose-stay group were required to press the lever that had not been presented in the information stage if reward had been given, but were required to press the lever that had been presented in the information stage if no reward had been given. Rats in the win-stay/lose-shift group were required to press the lever that had been presented in the information stage if reward had been given, but were required to press the lever that had not been presented in the information stage if no reward had been delivered. The choice stage was complete when the rat had made the required number of lever presses on one of the levers, which was the same as in the previous phase. The response requirement was identical to that in operation in the information stage. Both levers were then withdrawn. If the rat had chosen correctly, a food pellet was delivered and the tray light illuminated for 5 s, after which the ITI began. If the rat had chosen incorrectly, the levers were withdrawn but no food pellet was delivered and the ITI commenced. If the rat failed to complete the response requirement within 15 s, the levers were both withdrawn, the trial was abandoned, and the ITI commenced. Sessions lasted until the rat had completed 40 trials, or until 40 min had elapsed. There were 33 sessions of critical experimental training.

### Results and discussion

**Fig. 1:**
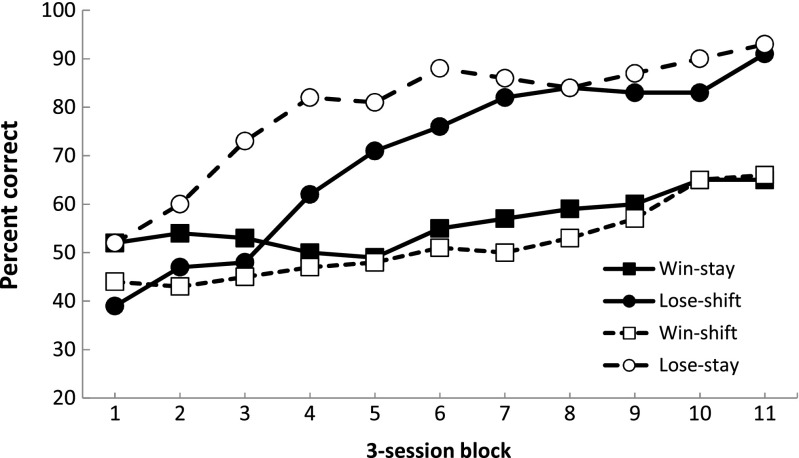
Mean percentage accuracy for each of the four reinforcement rules on each three-session block during Experiment 1. Solid shapes and complete lines are from the win-stay/lose-shift group, open symbols and dashed lines are from the win-shift/lose-stay group

The group-mean response accuracy for the four types of rule governing reinforcement delivery, represented as three-session blocks, are displayed in Fig. 1. The rats initially performed each of these contingencies at chance levels. Performance improved over the course of training, but did so at different rates depending on the rule experienced by the subjects. The win-shift/lose-stay group acquired the lose-stay rule more rapidly than the win-shift rule. The win-stay/lose-shift group acquired the lose-shift rule faster than the win-stay rule. Cross-group comparisons reveal that the lose-stay rule was learned most rapidly, followed by the lose-shift rule. By the end of training, both lose rules were performed at the same high level of accuracy. In contrast, the two win rules were acquired only slowly, with rats performing the win-stay rule, at least initially, with greater accuracy. However, neither win-based rule was attained to the same level of accuracy as either of the two lose-based rules.

An initial mixed-model analysis of variance (ANOVA) was conducted on these data with group (win-stay/lose-shift vs. win-shift/lose-stay) as a between-subject factor, and rule (win vs. lose) and block as within-subject factors. This analysis produced a significant three-way interaction between the factors, *F*(10,935) = 5.13, *p* < .01, *partial eta* ^*2*^ = .052. As the three-way interaction was significant, separate ANOVAs were conducted on the win-shift/lose-stay and win-stay/lose-shift groups. A repeated-measures ANOVA with reinforcement rule (win vs. lose) and block as factors was conducted for the win-shift/lose-stay group. This analysis revealed a significant main effect of rule, *F*(1,22) = 9.73, *p* < .01, *partial eta* ^*2*^ = .306, block, *F*(10,220) = 23.70, *p* < .001, *partial eta* ^*2*^ = .519, and an interaction between the two factors, *F* (10,220) = 6.32, *p* < .01, *partial eta* ^*2*^ = .223. Simple effect analyses conducted on the last block of training revealed that the lose-stay rule was performed at a greater accuracy than the win-shift rule, *F*(1,220) = 4.76, *p* < .05, *partial eta* ^*2*^ = .021. A two-factor ANOVA (rule × block) conducted on the win-stay/lose-shift group revealed a significant effect of rule, *F*(1,22) = 8.63, *p* < .001, *partial eta* ^*2*^ = .282, block, *F*(10,220) = 26.01, *p* < .001, *partial eta* ^*2*^ = .542, and an interaction between the factors, *F*(10,220) = 7.31, *p* < .001, *partial eta* ^*2*^ = .249. Simple effect analyses conducted on the last block of training revealed that the lose-shift rule was performed at greater accuracy than the win-stay rule, *F*(1,220) = 4.05, *p* < .05, *partial eta* ^*2*^ = .018.

An independent groups t-test conducted on the last block between the lose-stay and win-stay rules revealed that performance governed by the former rule was better than that governed by the latter, *t*(44) = 3.14, *p* < .01. The comparison between the lose-shift and win-shift rules on the last block also revealed performance according the lose rule was better than that according to the win rule, *t*(44) = 2.79, *p* < .01. The win-stay contingency was performed marginally better than the win-shift contingency on the last block, *t*(44) = 2.01, *p* < .06, but there was no difference between the two lose-based rules on the last block, *p* > .20.

The present results did not display the superiority of the win-shift compared to the win-stay contingency performance typically noted in mazes (see Cohen et al., 2004; Olton & Samuelson, 1976). Rather, using the training procedure often adopted for T-maze studies (e.g., Olton & Samuelson, 1976), the present study found, if anything, the typically noted superiority for win-stay relative to win-shift behavior (Morgan, 1974; Williams, 1991). It is not the case that win-stay rules have not previously been found to be learned in the conditioning chamber (e.g., Rayburn-Reeves et al., 2013), but the current results are novel in that they show these rules seems more readily acquired than a win-shift rule.

The current study did note that both of the win-based rules were learned only slowly relative to the two lose-based rules, which has not previously been noted. One factor that might have led to the poor performance according to the win rules might have been interference with the processing of the information stage outcome. Such interference may have been generated by the delivery of food and the illumination of the tray light at the termination of the information phase of the win trials. Non-target events presented during a retention period can impair performance on a variety of tasks, and it has been suggested that such attenuated performance is the product of a displacement of the target event (in this case the outcome of the information stage) from memory (e.g., Colwell, 1984; Wagner, Rudy, & Whitlow, 1973). If disruption of memory did occur, then performance according to both of the rules should be low relative to performance governed by the lose rules.

Alternatively, it is possible that, as rats were taught two rules simultaneously (i.e., either win-shift and lose-stay or win-stay and lose-shift), learning about one of the rules may have interfered with the acquisition of the other. It is possible that the subjects had an innate disposition toward performing according to one or other of the permutations studied. For example, rats in mazes appear to exhibit high levels of shift performance when not under contingency constraint (Cohen et al., 2004; Gittis, Stark, Arnold, Geter, Frazier, & Olton, 1988). Any such predisposition to one or other of the rules in the conditioning chamber might lead to behavior based on one rule interfering with that based on another.

## Experiment 2

Experiment 2 sought to replicate the findings from Experiment 1, and also to explore the above explanation of any advantage for the lose-based rules. The two possibilities discussed in Experiment 1 for better lose-based performance were addressed by requiring subjects to perform according to only one of the rules (win-stay, win-shift, lose-stay, or lose-shift). If the delivery of food following the information stage of a win trial disrupted performance, then acquisition of both win rules would be poor relative to the lose rules, even when the rules are taught in isolation. However, if there is interference from one rule overshadowing performance to another rule, then the pattern of results seen in Experiment 1 may not be replicated when the rules are taught separately.

### Method

#### Subjects and apparatus

Forty-eight experimentally naïve male Long Evans rats served in the present experiment. The subjects were 3–4 months old at the start of the experiment, had a free-feeding body weight of 250–350 g, and were maintained as described in Experiment 1. The apparatus consisted of four chambers described in Experiment 1.

#### Procedure

The subjects were magazine- and lever press-trained as described in Experiment 1. Following the pre-training phase, the subjects were split into four equal-sized groups (n = 12). Each group only received exposure to one of the four possible rules. One group received training on the win-shift rule, a second on the lose-shift rule, the third group on the win-stay rule, and the final group experienced the lose-shift contingency. The procedures for delivering these contingencies were as described in Experiment 1, with the exception that each group received only one type of rule. Sessions lasted until the rat had completed 40 trials, or until 40 min had elapsed. Training continued until the rats had reached a criterion of 80 % of trials correct for three successive sessions.

### Results and discussion

**Fig. 2:**
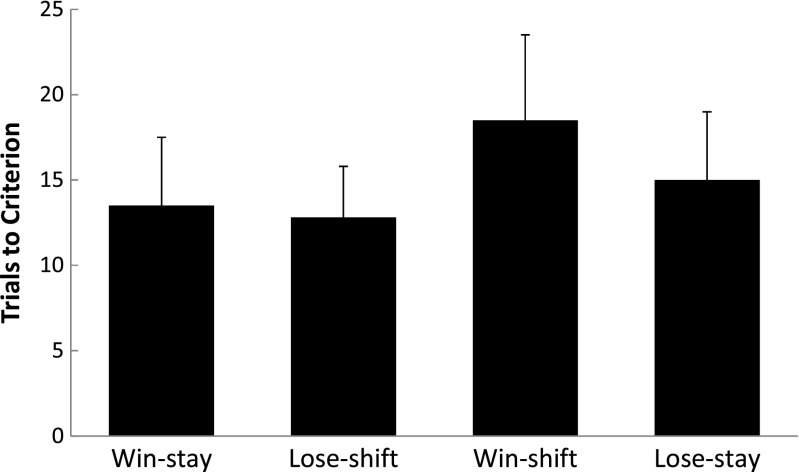
Group mean sessions to criterion for all four groups during Experiment 2. Error bars = 95 % confidence intervals

Figure 2 displays the group-mean number of sessions required to reach criterion performance for all four groups. The win-shift rule took more trials to reach criterion than each of the other three rules, which were all similar to one another. A between-subject ANOVA conducted on these data revealed a significant difference among the groups, *F*(3,44) = 8.07, *p* < .01, *partial eta* ^*2*^ = .355. Subsequent analyses of these differences by Tukey’s Honestly Significant Difference tests revealed that the win-shift rule was acquired less quickly than each of the other rules, all *p*s < .05. No other pairwise group comparisons were significant, all *p*s > .05.

That subjects acquired the win-stay rule, but not the win-shift rule, at a similar rate to the two lose-based rules suggests that, at least in the present procedure, the delivery of reinforcement and illumination of the tray light did not disrupt processing of the information preceding these events. If memory for the preceding events was impaired by these events, then performance of the win-stay and win-shift behavior would have been equally poor relative to the two groups experiencing the lose contingencies. These results are consistent with the suggestion that, in Experiment 1, learning about a lose-shift rule interfered with learning to perform according to a win-stay rule. Although it is not exactly clear on the basis of the present data why performance according to the lose contingencies should be acquired faster than performance governed by the win rules.

## General discussion

In both the present experiments, acquisition of a win-shift rule was not more rapid than the acquisition of any of the other the reinforcement rules studied, as might be suggested if spontaneous alternation behavior were the norm in the conditioning chamber. This pattern of results was obtained irrespective of whether the win-shift rule was taught in conjunction with a lose-stay rule (Experiment 1) or in isolation (Experiment 2). These findings from a procedure conducted in a conditioning chamber are at odds with those typically obtained in experiments that used apparatus such as the radial maze (see Dember & Richman, 2012 for an overview). However, they are consistent with those that have investigated this behavior in conditioning chambers (e.g., Morgan 1974; Rayburn-Reeves et al., 2013; Williams, 1991). Thus, although the present data confirm that rats may indeed learn a win-shift rule in an operant chamber (see Evenden & Robbins, 1984), they demonstrate that, when acquisition of a win-shift rule is compared to acquisition of other rules, the former rule is learned more slowly.

A novel finding that emerged from both of the present studies concerned the speed at which rats acquired the lose-stay rule. In both the present experiments, rats learned this reinforcement contingency rapidly. Neither the ecological view of spontaneous alteration (Olton, 1982), views based on the operation of molar maximizing principles (see Evenden & Robbins, 1984; Williams, 1991), nor a view of alternation behavior stemming from a simple version of the law of effect (see Williams, 1991), can supply a ready explanation for the high levels of performance of the lose-stay contingency. One view that may prove compatible with this result, however, can be derived from theories regarding the power of particular reward (e.g., Lea, 1981; Rachlin Battalio, Kagel, & Green, 1981). In broad terms, this approach suggests that the value of the reinforce (its power to maintain behavior) will be a function of the amount of “effort” (e.g., the number of responses) expended in order to obtained that reward. During lose trials the overall amount of reinforcement presented to the subjects is less than on win trials (i.e., a total of one instead of two reinforcers). This may tend to increase the value of the reinforce presented on those trials due to the greater number of responses needed to secure them, which, in turn, promotes learning of the lose contingencies. Such an account is speculative, but further investigation may serve to strengthen its integration into this area of reinforcement theory.

A potential method may be available by which to explore the above possibility, and was reported in a T-maze study by Cohen et al. (2004). In this study, each rule produced two reinforced trials and a single non-reinforced trial during forced-choice training with three rather two trial sequences. This would equate the level of reinforcement across the trials and allow investigation of the above suggestions. It should be noted, however, that the current results are unlikely to be explained solely by differences in the total amount of reinforcement present on the different types of trial – if that were the case, then the win trials, on which there is the possibility of more reinforcement, might be expected to produce faster learning than on the lose trials.

The reasons why spontaneous alternation occurs readily in the context of a maze study but in a conditioning chamber is currently unclear. It may be that the spatial aspects of the task, which may be more salient in a maze, favor the adoption of a win-shift strategy in this apparatus. A related issue is that the food reinforcement was not obtained at the location of the choice alternative in the operant chamber as it is in the maze. In the current procedure (but not in a T-maze) reinforcement is obtained from a common location (the food magazine) regardless of which lever is correctly pressed. The foraging-related explanations of win-shift tendencies tend to point to the fact that when food is obtained from a small patch in one location, it will not be available there again for some period of time. If this is true, then such theories might not predict win-shift superiority when the food is obtained somewhere other than the location(s) serving as the response alternatives. Alternatively, it may be that local feedback (i.e., what happened on the last trial) becomes more salient in the conditioning chamber for rats (Rayburn-Reeves et al., 2013) than in the maze, in which rats have been noted to chunk information about trial sequences (see Cohen et al., 2004). Another difference between the operant chamber and T-maze is that rats go to a location for food in a radial maze with no manipulation of food needed in the case of small pellets, which can be taken directly into the mouth. With bar press, rats manipulate an object to food. The bar might be conceived as an object, rather than a place. Such a dual system of place and object coding has been posited by Wang and Spelke (2002) in the context of human spatial cognition, and by Cheng (1986) in terms of “geometry in rats.” This distinction might matter in terms of which contingencies are most easily learned. Again further work will be needed to parse these possibilities.

In summary, the current studies found that rats will not learn a win-shift rule more readily than other forms of rule in the conditioning chamber. This finding was noted even when the same forced-choice procedure as used in T-maze studies was adopted. This suggests, at the very least, that the notion of a single inherent foraging strategy as complete explanation of rats’ tendencies to emit particular behavioural strategies will not explain all such performance.

## References

[CR1] Charnov EL (1976). Optimal foraging, the marginal value theorem. Theoretical Population Biology.

[CR2] Cheng K (1986). A purely geometric module in the rat's spatial representation. Cognition.

[CR3] Cohen J, Westlake K, Szelest I (2004). Effects of runway shift and stay rules on rats’ serial pattern learning in the T-maze. Animal Learning & Behavior.

[CR4] Colwill RM (1984). Disruption of short-term memory for reinforcement by ambient illumination. Quarterly Journal of Experimental Psychology.

[CR5] Dember, W. N., & Richman, C. L. (2012). *Spontaneous alternation behavior*. Springer Science & Business Media.

[CR6] Evenden JL, Robbins TR (1984). Win-stay behaviour in the rat. Quarterly Journal of Experimental Psychology.

[CR7] Gittis AG, Stark H, Arnold C, Beter B, Frazier D, Olton DS (1988). Emergence of choice strategies in the rat: shift-stay differentiation precedes win-lose differentiation. Animal Learning and Behavior.

[CR8] Krebs, J.R., & McCleery, R.H. (1984). Optimization in behavioural ecology.

[CR9] Lea SEG, Harzem P, Zeiler MD (1981). Correlation and contiguity in forgaing behavior. Advances in analysis of behavior (vol 2): Predictability, correlation and contiguity.

[CR10] Montgomery KC (1951). The relation between exploratory behavior and spontaneous alternation in the white rat. Journal of Comparative and Physiological Psychology.

[CR11] Morgan, M. J. (1974). Effects of random reinforcement sequences. *Journal of the Experimental Analysis of Behavior, 22*, 301–310. 10.1901/jeab.1974.22-301 PMC1333271 16811795

[CR12] Olton DS (1979). Mazes, maps, and memory. American Psychologist.

[CR13] Olton D (1982). Staying and shifting. Their effects on discrimination learning. Quantitative Analyses of Behavior.

[CR14] Olton DS, Collison C, Werz MA (1977). Spatial memory and radial arm maze performance of rats. Learning and Motivation.

[CR15] Olton DS, Samuelson RJ (1976). Remembrance of places passed: spatial memory in rats. Journal of Experimental Psychology: Animal Behavior Processes.

[CR16] Olton DS, Schlosberg PE (1978). Food-searching strategies in young rats: win-shift predominates over win-stay. Journal of Comparative and Physiological Psychology.

[CR17] Rachlin H, Battalio R, Kagel J, Green L (1981). Maximazation theory in behavioral psychology. Brain and Behavioural Sciences.

[CR18] Rayburn–Reeves RM, Stagner JP, Kirk CR, Zentall TR (2013). Reversal learning in rats (Rattus norvegicus) and pigeons (Columba livia): Qualitative differences in behavioral flexibility. Journal of Comparative Psychology.

[CR19] Stanton MS, Thomas GJ, Brito GN (1984). Posterodorsal septial lesions impair performance on both shift and stay working memory tasks. Behavioral Neuroscience.

[CR20] Timberlake W, White W (1990). Winning isn't everything: Rats need only food deprivation and not food reward to efficiently traverse a radial arm maze. Learning and Motivation.

[CR21] Wagner AR, Rudy JW, Whitlow JW (1973). Rehearsal in animal conditioning. Journal of Experimental Psychology.

[CR22] Wang RF, Spelke ES (2002). Human spatial representation: Insights from animals. Trends in cognitive sciences.

[CR23] Williams BA (1991). Choice as a function of local versus molar reinforcement contingencies. Journal of the Experimental Analysis of Behavior.

